# Spurious Elevations of Chromogranin A in the Setting of Autoimmune Metaplastic Atrophic Gastritis

**DOI:** 10.7759/cureus.102050

**Published:** 2026-01-22

**Authors:** Elizabeth Jose, Ariana Ambrosio, Jesse F Simon, Jeffrey H Schneider

**Affiliations:** 1 Internal Medicine, Nova Southeastern University Dr. Kiran C. Patel College of Osteopathic Medicine, Davie, USA; 2 Internal Medicine, Kansas City University, Kansas City, USA; 3 Radiology, Nova Southeastern University Dr. Kiran C. Patel College of Osteopathic Medicine, Davie, USA; 4 Gastroenterology, Broward Health Coral Springs, Coral Springs, USA

**Keywords:** autoimmune metaplastic gastritis, chromogranin a, enterochromaffin cell hyperplasia, gastric neuroendocrine tumors, gastritis, gastroenterology, gastrointestinal, hypergastrinemia, neuroendocrine tumors, pernicious anemia

## Abstract

Autoimmune metaplastic gastritis (AMAG) is a form of autoimmune gastritis that is characterized by the immune system’s attack on gastric parietal cells, leading to chronic inflammation. Gastric neuroendocrine tumors (GNETs) are rare neoplasms that can develop in the gastrointestinal tract in the presence of AMAG. This case presents a 69-year-old female who presented with dyspepsia, and on subsequent endoscopic evaluation, she was found to have AMAG in the context of elevated levels of serum chromogranin A (CgA) and gastrin, suggestive of a GNET. Despite an extensive diagnostic workup, including imaging, colonoscopy, small bowel follow-through, capsule study, and oncology workup, no GNET was identified. She was also found to have antibodies to parietal cells, suggestive of pernicious anemia. The elevated markers were attributed to enterochromaffin cell hyperplasia, secondary to hypergastrinemia from AMAG. This case invites a discussion about the need for more evidence-based guidelines in the workup and monitoring of spurious elevations of CgA and gastrin in the presence of AMAG. It also highlights the importance of careful and intentional clinical evaluation to avoid unnecessary tests and costs to the patient.

## Introduction

Gastric neuroendocrine tumors (GNETs) are rare neoplasms within the gastrointestinal tract that can develop in the setting of autoimmune gastritis (AIG) [1]. Autoimmune metaplastic atrophic gastritis is a specific form of AIG observed in the absence of gastric parietal cells, leading to hypergastrinemia due to hypochlorhydria and therefore resulting in hyperplasia of enterochromaffin-like (ECL) cells [1]. This chronic inflammatory disease is uncommon in gastroenterology patients and disproportionately affects elderly women [2]. The diagnoses of AIG and GNETs are usually associated with nonspecific symptoms and are frequently discovered incidentally during gastric endoscopies [3]. Endoscopies with biopsies are the recommended method for detecting and monitoring gastric cell changes [4]. Serum chromogranin A (CgA) can be a valuable but nonspecific marker in the monitoring of GNETs as well as the progression of AIG [3]. CgA is a glycoprotein found in neuroendocrine cells and can be elevated in the presence of ECL cell hyperplasia and serum gastrin levels [3]. Reports have shown a strong correlation between serum CgA and gastrin, although CgA does not necessarily correlate with ECL density [4]. In addition to malignancy, vitamin B12 deficiency and pernicious anemia are long-term consequences of AIG [2]. The most common cause of vitamin B12 deficiency is pernicious anemia, which accounts for 20-50% of cases with vitamin B12 deficiency and results from atrophic gastritis [5]. It has been reported that GNETs developing from ECL cells hyperplasia induced by chronic hypergastrinemia can occur in 4-9% of patients with a history of AIG and pernicious anemia [6]. In this report, we present a patient with a presumed neuroendocrine tumor based on spurious elevations of CgA and gastrin as well as findings of atrophic gastritis during an endoscopy.

## Case presentation

Herein is the review of a case of a 69-year-old female with a past medical history including gastroesophageal reflux disease (GERD), small hiatal hernia, and esophagitis. The patient presented to the gastroenterologist with the complaint of dyspepsia that worsened with stress, and palpitations (followed by cardiology).

**Figure 1:**
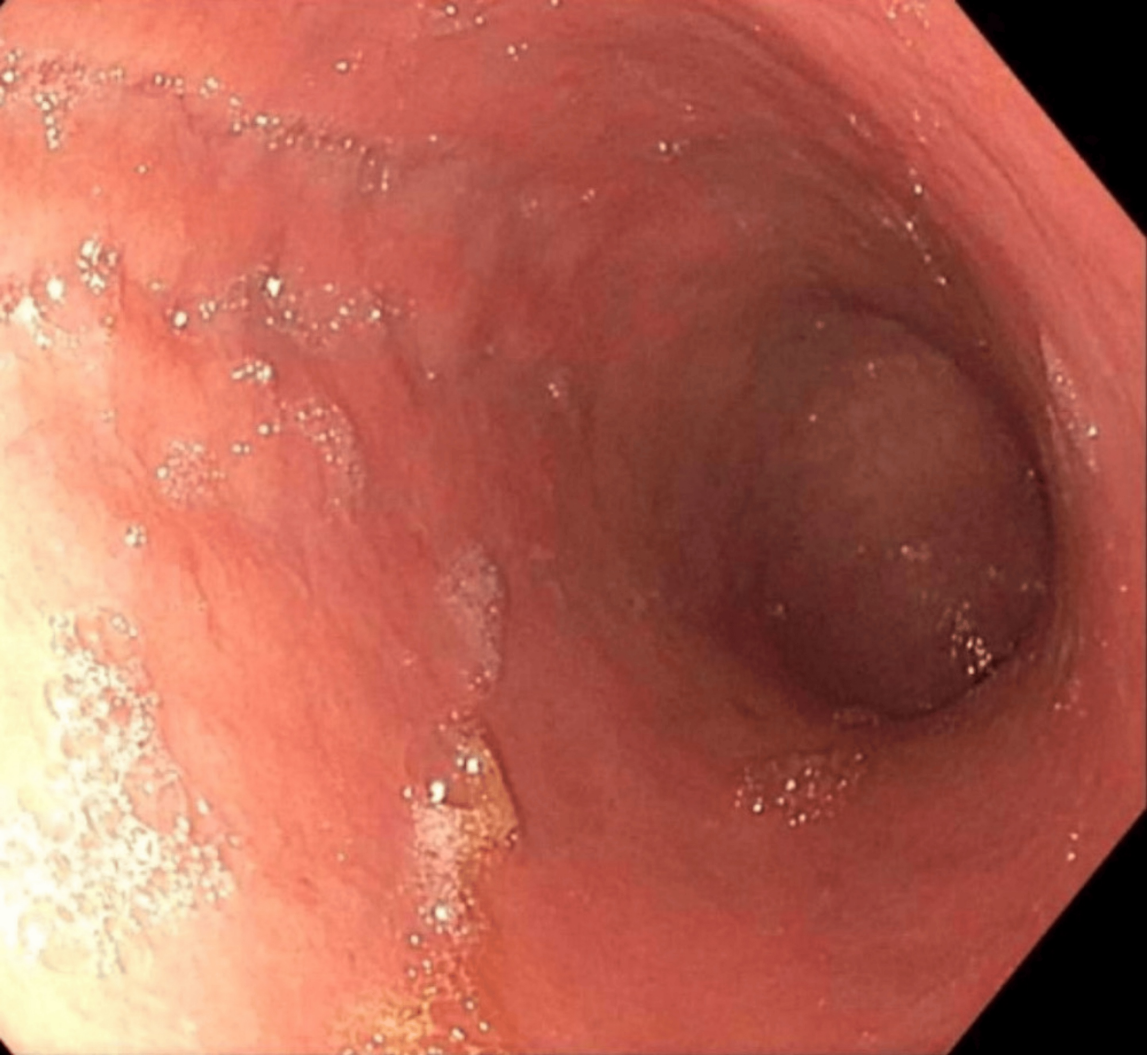
Endoscopic view of gastric body with patchy inflammation

**Figure 2:**
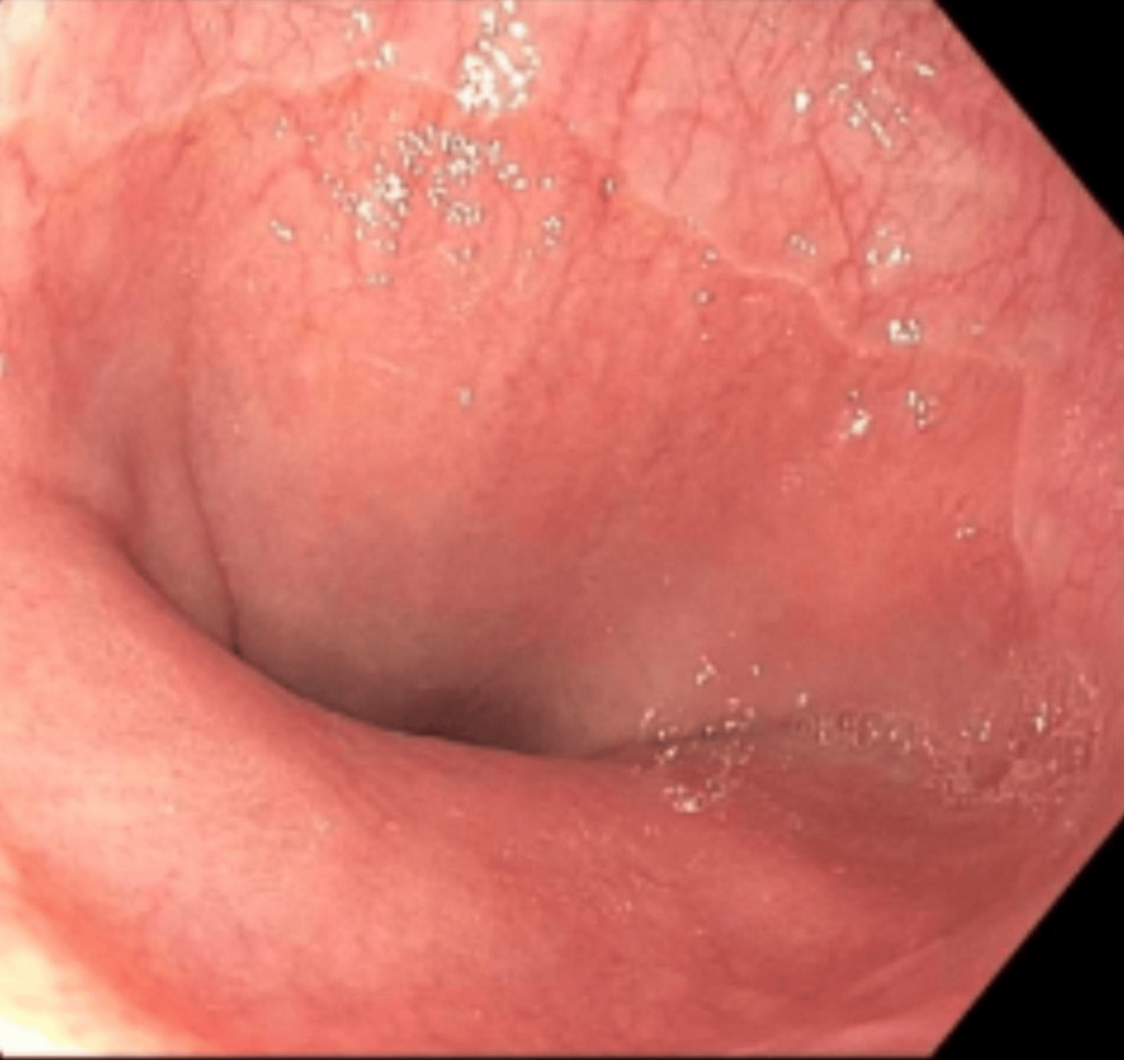
Endoscopic view of normal gastroesophageal junction

On upper endoscopy, there was noted patchy minimal inflammation in the entire stomach (Figure 1) and a normal esophagus (Figure 2) and duodenum. Biopsies from the upper endoscopy showed chronic active gastritis with intestinal metaplasia and atrophic features suggestive of AMAG. A further evaluation as part of a workup for her AMAG recommended by the pathologist, revealed a elevated CgA level of 812 ng/mL (NML < 39ng/ml), an elevated gastrin level of 3115 pg/mL (NML 0-180 pg/ml), a parietal cell antibody titer positive at >1:64 H (NML - <1:20), and Vitamin B12 390 pg/mL (200-1100) (Table 1). A previous colonoscopy noted diverticulosis and an unremarkable terminal ileum and colon.

**Table 1:** Relevant laboratory findings in evaluation for autoimmune metaplastic atrophic gastritis and suspected neuroendocrine tumor 5-HIAA: 5-hydroxyindoleacetic acid; LDH: lactate dehydrogenase

Test	Result	Reference
Chromogranin A (Initial)	812 ng/mL	< 39 ng/mL
Chromogranin A (Follow-Up)	569 ng/mL	< 39 ng/mL
Gastrin	3115 pg/mL	0-180 pg/mL
Parietal Cell Antibody Titer	>1:64 H	< 1:20
Vitamin B12	390 pg/mL	200-1100 pg/mL
24-Hour Urine 5-HIAA	Normal	< 6.0 mg/24hr
White Blood Cell Count	3.60 x 109/L	4.0 - 10.5 x 109/L
Hemoglobin (Hgb)	11.3 g/dL	12.0-15.5 (Female) g/dL
LDH	Normal	140-280 U/L

She was subsequently referred to oncology to discuss her recent diagnosis and undergo an evaluation for a possible carcinoid tumor. At a later consultation, she underwent an evaluation that noted a normal liver profile and lactate dehydrogenase (LDH). She was noted to have a chromogranin A level of 569 ng/ml. A urine 24H collection of 5HIAA (5-hydroxyindoleacetic acid) was normal. A clinical differential diagnosis of a neuroendocrine tumor (NET) with potential Zollinger-Ellison syndrome and enterochromaffin cell hyperplasia (ECH) was considered.

During consultation with oncology, the patient complained of chronic dyspepsia but denied having chronic diarrhea, urticarial skin rashes, chronic wheezing, unintentional weight loss, palpable adenopathy, or bone pains. She complained of intermittent palpitations. Additional labs by oncology showed: WBC 3.60 and Hgb 11.3 g/dL. The patient was previously prescribed proton pump inhibitors, but endorsed that she only took them for a brief period and had not taken them for at least two weeks prior to obtaining serum gastrin and CgA levels. She noted taking famotidine and Mylanta as needed for GERD. She had no history of peptic ulcer disease. She then underwent a series of diagnostic studies to find the presumed neuroendocrine tumor (NET) as the source of elevated CgA. A Gallium-68 Dotatate CT/PET scan was requested as part of this evaluation, but was subsequently denied by the patient’s insurance carrier. Oncology reported that she most likely has enterochromaffin cell hyperplasia secondary to hypergastrinemia, which elevated serum CgA level. She was then recommended to follow up with her GI specialist, at which time a small bowel follow-through series and a Pill Cam study were both negative. A repeat colonoscopy and ileoscopy were also negative. No GNET was ever found throughout the workup.

The patient is currently being treated with sublingual Vitamin B12 1 mg orally once a day. She is also currently scheduled for regular follow-up with her gastroenterologist for continual monitoring and treatment of autoimmune gastritis, pernicious anemia, CgA levels, and gastrin levels. She was educated on the signs and symptoms of NET and instructed to call the office for an appointment if she began experiencing any symptoms.

## Discussion

This case report provides an opportunity to review the correlation of elevated serum levels of CgA in the setting of AMAG and GNETs. This patient's lab values match other reports of GNETs in that the patient has increased levels of CgA and gastrin, underlying B12 deficiency, and the endoscopy report indicated active gastric inflammation. However, despite extensive workup, no GNET was discovered. 

AIG is a premalignant condition due to autoimmune destruction of gastric parietal cells [2]. The World Health Organization divides GNETs into three categories. Type 1 tumors are linked to hypergastrinemia and are typically due to chronic AIG [1]. While type 2 GNETs develop secondary to gastrinoma or Zollinger-Ellison syndrome [1]. Both type 1 and type 2 tumors are generally associated with a favorable overall prognosis [1].

Of reported cases of neuroendocrine tumors, the average serum CgA levels were greater than 200 ng/ml and gastrin levels greater than 500 ng/ml [6,7]. Our patient had an initial serum CgA level of 812 ng/ml and a gastrin level of 3115 ng/ml. The study conducted by Kalkan et al. found the highest serum CgA level in patients with carcinoids was 733 ng/m but suggested that CgA values may just be elevated in patients due to the presence of AIG without the presence of a GNET [3]. Through further investigation, they recommended that while assessing elevated CgA results in patients with AIG, other factors influential to CgA should be accounted for, such as the presence of corpus atrophy, positivity of Helicobacter pylori immunoglobulin G (IgG), and the presence of ECL cell hyperplasia [3].

This patient had active chronic gastritis with intestinal metaplasia and atrophy with a positive antibody titer for parietal cells. Pernicious anemia can result from autoimmune destruction and the progressive loss of parietal cells in the stomach seen in AIG [5]. Diagnostic markers of pernicious anemia involve the presence of autoantibodies to gastric parietal cells, achlorhydria, and elevated serum levels of gastrin and chromogranin A [5]. This previous study indicated a high prevalence of gastric body atrophic gastritis, premalignant and malignant stomach lesions in patients with pernicious anemia [5].

A 2023 systematic review by Chen et al. sought to determine the relative risk of gastric neoplasms arising in the presence of diagnosed autoimmune metaplastic atrophic gastritis [8]. Meta-analysis showed a relative risk of 11.05 (95% CI: 6.39-19.11) for gastric cancer development in AMAG patients. Further, there was an incidence of 0.52% and 0.83% per AMAG patient per year of gastric low-grade dysplasia and type-1 gNETs [8]. A study conducted by Mahmud et al. determined there was a high prevalence and incidence of gastric cancer in association with AMAG and therefore recommended endoscopic surveillance in all patients with AMAG, especially in patients older than 70 years old [9]. Our findings parallel current guideline approaches, which recognize AIG as a premalignant condition and support endoscopic surveillance.

As seen in this case, there is also a matter of cost burden to the patient that should be considered. The insurance of the patient denied the coverage of the Gallium-68 Dotatate CT/PET, which was meant to investigate for a possible NET. It is also prudent to consider further risks to the patient due to over-testing. As seen by this case and past cases, there is variation in how clinicians decide to investigate elevated CgA and gastrin. Further guidelines should caution clinicians that elevated CgA levels don’t necessarily signify potential GNET; therefore, clinicians should use clinical judgment in evaluating a patient for GNET before proceeding with an extensive workup.

## Conclusions

In this case report, the spurious increased levels of CgA and gastrin in the setting of AMAG and underlying B12 deficiency led to an extensive workup in search of a potential neuroendocrine tumor, which did not exist in this patient. Our case is atypical in that the GNET screening found no tumor despite what the literature indicates. Therefore, since similar cases could occur, this case report highlights the importance of considering that spurious elevated levels of CgA and gastrin in the setting of AMAG and B12 deficiency may not actually result in a GNET.
